# Children and adolescents with sickle cell disease: characteristics and use of medicinal plants

**DOI:** 10.1590/1984-0462/2025/43/2023262

**Published:** 2024-10-04

**Authors:** Rebeca Garcia de Paula, Hewerthon Medrado Ribeiro, Luciana de Melo Borges, Lucas Barbosa e Souza, Kellen Lagares Ferreira Silva, Carla Simone Seibert

**Affiliations:** a Universidade Federal do Tocantins, Palmas, TO, Brasil.

**Keywords:** Sickle cell disease, Pediatrics, Medicinal plants, Doença falciforme, Pediatria, Plantas medicinais

## Abstract

**Objective:**

The aim of this study was to describe the epidemiological characteristics of pediatric patients with sickle cell disease (SCD) and evaluate the use of medicinal plants in these patients concomitantly with their drug treatment.

**Methods:**

This is a review of the medical records of pediatric patients at a public institution with tertiary care. The selection criterion was to be a child or adolescent with SCD undergoing pediatric follow-up at this outpatient clinic. In the medical records of the patients selected, records of the use of plants for medicinal purposes were sought.

**Results:**

In total, 154 records (100% of total active patients followed in this clinic) were reviewed: 99 children and 55 adolescents. The predominant genotype was SS (58.4%), followed by SC (29.2%). The use of at least one medication for SCD was reported in 95.5% of the medical records. The use of medicinal plants was reported by 70.1% of patients, with 276 citations in the medical records referring to 64 different types of plants. Six plants were used for the treatment of SCD, the main one being Lemonvine/*Ora-pro-nóbis* (*Pereskia aculeata*). The use of medicinal plants was reported for flu-like symptoms and/or COVID-19 (both for prevention and treatment) by 60.5% of the citations, with 35 different plants reported for this use, exclusively or not. This use was followed by pain symptoms (14.2% of citations).

**Conclusions:**

The majority of patients in this study use both conventional and traditional/complementary/alternative medicine, highlighting the need for more studies in the area, with a special focus on patient safety.

## RESUMO

**Objetivo:**

Descrever as características epidemiológicas de pacientes pediátricos com doença falciforme (DF) e avaliar o uso de plantas medicinais concomitantemente ao tratamento medicamentoso.

**Métodos:**

Realizou-se revisão de prontuários de pacientes pediátricos de uma instituição pública com nível de atendimento terciário; o critério de seleção foi ser criança ou adolescente com DF em acompanhamento pediátrico neste ambulatório. Nos prontuários dos pacientes selecionados foi procurado o registro do uso de plantas com finalidades medicinais.

**Resultados:**

Foram revisados 154 prontuários (100% dos pacientes ativos desse ambulatório): 99 crianças e 55 adolescentes. O genótipo predominante foi SS (58,4%), seguido do SC (29,2%). O uso de ao menos uma medicação para a DF foi relatada em 95,5% dos prontuários. Em 70,1% dos prontuários havia o relato do uso de plantas medicinais, em 276 citações, sendo 64 plantas citadas ao todo; destas, seis espécies foram citadas para o tratamento da doença de base (DF), sendo “ora-pro-nobis” (*Pereskia aculeata*) a principal. Das 276 citações de plantas medicinais nos prontuários, 60,5% incluíram o uso de plantas medicinais em sintomas gripais e/ou COVID-19 (tanto prevenção quanto tratamento), sendo relatadas 35 para este uso, exclusivamente ou não. Essa foi a principal finalidade de uso encontrada nos prontuários dos pacientes, seguida do uso para sintomas de dor (14,2% das citações).

**Conclusões:**

Notou-se que a maioria dos pacientes avaliados faz uso da medicina convencional, aliada à tradicional/ complementar/ alternativa, o que leva à necessidade de mais estudos nessas áreas, especialmente na pediatria, visando principalmente à segurança do paciente.

## INTRODUCTION

Sickle cell disease (SCD) is a group of hemoglobinopathies with hereditary and autosomal characteristics, including various genotypes, in which hemoglobin S (Hb S) predominates in the erythrocytes.^1,2^

There are no specific studies on the pediatric population with SCD in some Brazilian areas, including Tocantins. There are, however, diagnostic studies in quilombo communities in this state, due to the African origin of the disease, identifying a significant number of people with sickle cell trait.^3,4,5^ It is known that SCD has a high prevalence in Brazil and, according to the Clinical Protocol and Therapeutic Guidelines for the Disease, it has been estimated to affect between 60,000 and 100,000 patients in the country. It is also known that in the northern region of Brazil there is a high incidence of the disease, while historically such families have also had a low socioeconomic level.^6^ The incidence of newborns diagnosed with SCD for every 10,000 live births in Tocantins ranged from 1.57 to 4.09 during the period 2016–2019, according to the Annual Reports of the National Newborn Screening Program/Live Birth Information System (SINASC). In 2022, there were 737 patients with SCD registered with the Hemovida Web Hemoglobinopathies System (SHWH) in the state of Tocantins, 291 of whom were aged between 0 and 18 incomplete years, according to data from the Ministry of Health/General Coordination of Blood and Blood Products (personal communication).

Children and adolescents with SCD need special care, given the particularities of the clinical manifestations, especially the high mortality rate under the age of three, usually due to an infectious event if there is no adequate follow-up. These patients need preventive measures, such as early diagnosis, follow-up with a pediatrician, family education, the use of special vaccines and prophylactic antibiotics, treatment of complications, and prevention of chronic organ damage.^2,7^

There are studies on using medicinal plants in SCD, especially in Africa. There are also systematic reviews and patents on phytotherapeutic drugs. Nevertheless, more research is needed in the areas in Brazil, including in the North, especially in the pediatric population.^8^

Therefore, the purpose of this study was to describe in detail the epidemiological characteristics of the population of pediatric sickle cell patients in Tocantins and to investigate whether this group of patients use medicinal plants.

## METHOD

This is a documentary study, with a quantitative approach, using descriptive methodology with a review of medical records. The data were collected from June 2022 to May 2023, in the city of Palmas, capital of the state of Tocantins, at the Hematology Outpatient Clinic of the Blood Network, where patients with SCD in Tocantins are monitored by the Unified Health System (SUS) and followed by a multi-professional team. Among 321 pediatric patients (children and adolescents) in this outpatient clinic, 183 (57%) present SCD.

The study population consisted of children aged 0 to under 12 years and adolescents aged 12 to under 18 years, who were being followed up by a pediatrician at the clinic, which were the inclusion criteria for this study. Pediatric patients (children and adolescents) who had not attended appointments for more than 2 years, and whose medical records had been archived, were excluded from the analysis (exclusion criterion for this study).

In these medical records, information on the use of medicinal plants (the name of any plant and/or natural substances) in the family treatment of these patients was researched, as well as their clinical and laboratory evolution, including the reports filed by the entire multi-professional team in their follow-up. Such information is routinely noted in the medical record by members of the Outpatient Multidisciplinary Team, in which Pediatrics is included. Each medication or plant described with a medicinal purpose was considered a citation, and each medical record represented a patient.

These medical records were reviewed from their inception to the day they were analyzed. To this end, the research was approved by the Research Ethics Committee (CEP) of the Federal University of Tocantins (UFT), under the consolidated decision 5.154.839 and CAAE: 51986621.2.0000.5519.

For this review of medical records, the form of the Hemoglobinopathies Outpatient Clinic of the Clinical Hospital of the Federal University of Goiás was adapted to guide the analysis of medical records (type of SCD, comorbidities, medications in use, transfusions, complementary tests, complications, vaccinations, investigation of the disease in the heel prick test, blood type, baseline hemoglobin, and Hb electrophoresis, among other tests). The guide also included questions about family income, whether they receive sickness benefits, the number of people living in the household, housing conditions, school performance, physical activity, seasonality of symptoms in relation to climate transition, stress factors, and complications during the pandemic (COVID 19), among others.

## RESULTS

A total of 154 records were reviewed at the Hematology Outpatient Clinic (100.0% of the active records of pediatric patients), totaling 99 children (aged 0 to under 12 years; mean age: 6.7 years, median age: 7 years) and 55 adolescents (aged 12 to under 18 years; mean age: 14.4 years, median age: 15 years) (Table 1). With regard to their state of residence, 151 lived in Tocantins, one in Pará, one in Goiás, and one in Mato Grosso. As for where they came from, these patients came from 48 different municipalities, the capital Palmas being the main one (42 patients; 27.3% of the total).

**Table 1:** Review of medical records of pediatric sickle cell patients at the Hematology Outpatient Clinic of the Blood Center of Palmas (TO). Data were collected from June 2022 to May 2023.

	Child 0–12 years old	Adolescent 12–18 years old	n	%
Sex				
Masculine	54	24	78	50.6
Feminine	45	31	76	49.4
Diagnosis				
Electrophoresis	40	48	88	57.1
Neonatal screening	59	07	66	42.9
Genotype				
SS	60	30	90	58.4
SC	30	15	45	29.2
Others	09	10	19	12,4
Race				
Mixed	84	46	130	84.4
Black	09	07	16	10.4
White	06	02	08	5.2
Blood type				
O +	49	33	82	53.2
A +	30	12	42	27.3
B +	10	04	14	9.1
O -	03	04	07	4.5
AB +	04	02	06	3.9
A -	02	00	02	1.3
Not tested	01	00	01	0.6
Clinical complications				
Pain crisis	52	30	82	53.2
Infections	39	14	53	34.4
Biliary lithiasis	10	07	17	11.0
Acute thoracic syndrome	05	01	06	3.9
Splenic sequestration	04	01	05	3.2
Splenectomy	01	02	03	1.9
Stroke	01	01	02	1.3
Priapism	01	00	01	0.6
Transfusions				
No	82	54	136	88.3
Yes	17	01	18	11.7
Vaccines up to date				
Yes	55	22	77	50.0
No	44	33	77	50.0
Sickness benefit (chronic disease)				
Yes	43	21	64	41.6
No	44	17	61	39.6
Not informed	12	17	29	18.8
Interference of weather/climate				
Yes	50	24	74	48.1
No	29	06	35	22.7
Not informed	20	25	45	29.2
Use of medicinal plants				
Yes (reported use)	70	38	108	70.1
No (use denied)	22	08	30	19.5
Not informed	07	09	16	10.4

At the last visit recorded in the medical records of the population studied, 77 (50.0%) patients had a vaccination schedule that had not yet been updated, taking into account both the National Immunization Program and the special vaccines for SCD, as established by the Reference Center for Special Immunobiologicals. The non-mandatory vaccines that are only available in the private network, according to the guidelines of the Brazilian Societies of Pediatrics and Immunology, were not considered for the “not updated” vaccination situation (vaccination delay) in the medical records (Table 1).

In 74 medical records, there were reports of pain crises related to changes in temperature, mainly to the cold, either due to changes in environmental temperature or to conditions generated by the cooling of the body after a cold water/river bath (gusts of wind after bathing). In 45 (29.2%) medical records, this information was not recorded, and 35 patients/guardians (22.7%) did not report noticing symptoms with changes in weather/climate (Table 1). These situations (weather/climate changes) were reported in some medical records as also triggering diseases related to the respiratory system, such as coughing (5; 6.7%), flu (4; 5.4%), allergies (rhinitis, sinusitis, asthma: 3; 4.0%), sore throat (1; 1.3%), and pneumonia (1; 1.3%).

With regard to medications, the 154 medical records analyzed recorded 400 instances of medication used by pediatric patients during their last visit (Table 2). In relation to SCD, among the 154 patients, 147 (95.5%) reported using folic acid; in contrast, only 20 (13%) were using hydroxyurea.

**Table 2:** Medications recorded in the last consultation in the medical records of pediatric sickle cell patients at the Hematology Outpatient Clinic of the Blood Center of Palmas (TO). Data were collected from June 2022 to May 2023.

Medications	n	%*	%^†^
Folic acid	147	36.8	95.5
Dipyrone	68	17.0	44.2
Phenoxymethylpenicillin	34	8.5	22.1
Paracetamol	28	7.0	18.2
Hydroxyurea	20	5.0	13.0
Ibuprofen	9	2.3	5.8
Tramadol	9	2.3	5.8
Morphine	7	1.8	4.5
Nimesulide	6	1.5	3.9
Albendazole	5	1.3	3.2
Captopril	5	1.3	3.2
Ceftriaxone	4	1.0	2.6
Acetylsalicylic acid	4	1.0	2.6
Vitamin D	3	0.8	1.9
Clarithromycin	3	0.8	1.9
Buscopan	3	0.8	1.9
Hydrocortisone	3	0.8	1.9
Amoxicillin + clavulonate	3	0.8	1.9
Others^‡^	39	9.8	-
**Total**	400*	100	-

*In relation to the number of medications reported in the last consultation;

^†^In relation to the number of medical records analyzed;

^‡^Frequency ≤2.

Table 3 ^8,9,10,11^ describes which plants were mentioned in the medical records analyzed, their purpose, form of use, and common and scientific names.^9,10,11^ A total of 64 medicinal plants were reported in 276 citations, because a single medical record could cite more than one medicinal plant, reported by the patient or their family member.

**Table 3:** Use of medical plants reported in the medical records of pediatric sickle cell patients at the Hematology Outpatient Clinic of the Blood Center of Palmas (TO). Data were collected from June 2022 to May 2023^8,9,10,11^.

Common names (English, when available/Portuguese)	Scientific name*	Purpose	Forms of use	n^†^	%
1. Spearmint/*Hortelã/menta/vick* ^‡^	*Mentha spicata*	Flu, flu prevention, abdominal pain, fever, gastrointestinal symptoms, COVID-19 Prevention, lower back pain, massage for pain	Tea, syrup, gel	31	11.2
2. Turmeric/*Açafrão*	*Curcuma longa*	Flu, abdominal pain, COVID-19	Tea, syrup, treacle	30	10.9
3. Lemon balm/*Erva cidreira* ^c^	*Melissa officinalis*	Flu, fever, calming, abdominal pain, lower limb pain, immunity, inflammation	Tea	21	7.6
4. Lemon/*Limão* ^c^	*Citrus limon/ Citrus aurantiifolia*	Flu, flu prevention, COVID-19, COVID-19 prevention	Tea, syrup	20	7.2
5. Garlic/*Alho*	*Allium sativum*	Flu, COVID-19, COVID-19 prevention, jaundice	Tea, syrup	16	5.8
6. Ginger/*Gengibre*	*Zingiber officinale*	Flu, COVID-19 prevention, COVID-19, sore throat	Tea, syrup	15	5.4
7. Lemongrass/*Capim-santo/capim-de-cheiro*	*Cymbopogon citratus*	Flu, immunity, COVID-19, COVID-19 prevention, fever, calming, abdominal pain	Tea	11	4.0
8. Blue spur flower/*Boldo* ^‡^	*Peumus boldus/Plectranthus barbatus*	Gastrointestinal symptoms, abdominal pain, jaundice, flu	Tea	8	2.9
9. Others^§^	^§^	^§^	^§^	124	44.9
Total				276	100.0

*Probable scientific names related to the common names of the medicinal plants reported;

^†^Number of citations reported in the medical records;

^‡^Not reported in the international literature on medicinal plants used in the treatment of SCD;

^§^Species with six or fewer citations (see Appendix 1 available with authors).

Six medicinal plants were cited for the underlying disease (“sickle cell anemia/sickle cell disease”), with four reports of the use of lemon vine/*Ora-pro-nóbis*, two of the use of cricket vine/*Pariri*, one of custard apple/*Pinha*, one of annatto/*Urucum*, one of onion/*Cebola,* and one of “*Casca de pau”* (unidentified species).

Of these 276 citations of medicinal plants in these 154 medical records, 136 (60.5%) included the use of medicinal plants for flu-like symptoms and/or COVID-19 (both prevention and treatment), with 35 medicinal plants reported for this use, exclusively or not. Flu-like symptoms were the main reason for using medicinal plants, followed by pain (32 citations; 14.2%), in the medical records studied (Tables 3 and 4).

**Table 4:** Purposes of the use of medicinal plants reported in the medical records of pediatric sickle cell patients at the Hematology Outpatient Clinic of the Blood Center of Palmas (TO). Data were collected from June 2022 to May 2023.

Use (medical records)	n*	%
Influenza	96	42.7
Pain	20	8.9
COVID-19 prevention	17	7.6
Influenza prevention	13	5.8
Gastrointestinal symptoms	12	5.3
Abdominal pain	12	5.3
Jaundice	10	4.4
COVID-19	10	4.4
Fever	8	3.6
Sickle cell disease	6	2.7
Insomnia	4	1.8
Calming and anti-anxiety	4	1.8
Immunity	3	1.3
Blood purification	2	0.9
Dengue fever	2	0.9
Anemia	1	0.4
Urinary symptoms	1	0.4
	225	100.0

*Number of medical records citing this use.

In addition to plants, other natural products were mentioned: 44 associations with honey and two with propolis, four with “tea with alcohol/bottled infusion,” three with “grandma’s syrup,” one with “snake venom (rattlesnake),” one with ostrich oil, one with chicken lard, and one with essential oils, among others.

**Figure 1:**
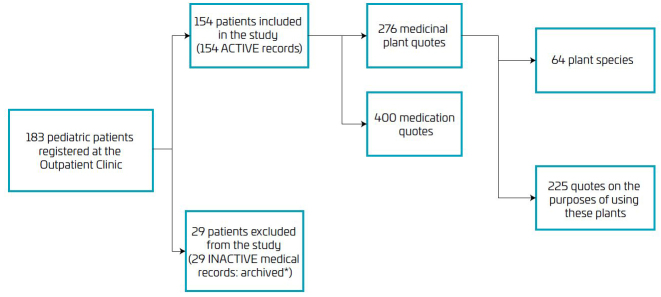
Flowchart of the medical records of pediatric sickle cell patients at the Hematology Outpatient Clinic of the Blood Center of Palmas (TO). Data were collected from June 2022 to May 2023.

A total of 29 pediatric patients (13 children and 16 adolescents) had not been to their appointments for more than 2 years and their medical records had been archived, so they were excluded from the analysis in this study. In total, 183 patients aged 0 to 18 years with SCD were registered with the Ministry of Health’s Webhemoglobinopathies System for the Hematology Outpatient Clinic of the Blood Center of Palmas on the last day of the medical records search (May 31, 2023) (Figure 1).

*non-attendance at the Outpatient Clinic for more than 2 years

## DISCUSSION

This study provides evidence of the widespread use of medicinal plants in the population of patients with SCD in developing areas, such as the state of Tocantins, places that may resemble the vast majority of Brazil and even other parts of the world. The population studied uses medicinal plants, but their use is not necessarily supported scientifically or in synergism with the practices of the health professionals who accompany them. This is in contrast to some studies, which have already outlined some natural products with in vitro and in vivo effects for SCD, thus opening up a large field for research, even more so in a context where the use of hydroxyurea is precarious, despite it being the most important drug for treating SCD.^8^

In the population studied, patients with hemoglobin SS predominated, followed by hemoglobin SC, which is in line with the prevalence of the S and C genes in the population of Tocantins.^3,4,5,6^

The heel prick test (neonatal screening) indicated the diagnosis in 42.9% of the patients evaluated in the review of medical records, and 57.1% received the diagnosis only after the neonatal period, which is in line with what is expected for the population of the age group analyzed, since only after 2013 were hemoglobinopathies included in the neonatal screening carried out by the Unified Health System (SUS) in Tocantins and, according to Law No. 14.154, of May 21, 2021, only stage 1 is already covered in the state. Another four stages still need to be implemented, so that more diseases can be screened for.^12^

The results regarding the patients’ vaccination delay are in line with studies on low vaccination coverage in the state and the country, especially after the COVID-19 pandemic.^13^ They are corroborated by the international literature, with parents being particularly wary of vaccinating their children.^14^ Such patients are more susceptible to infection and, consequently, to hospitalization, so this is an additional reason for them to keep their vaccination schedule up to date.^2^

Possible reasons for these drops, and especially for the fear of getting vaccinated against COVID-19, include the following: anti-vaccine movements, “fake news,” inefficient communication from health professionals, and the opening hours of vaccine rooms at health centers (usually during business hours, when those responsible for pediatric patients are working), among others. On the other hand, the decision to get vaccinated requires a favorable environment, social influences, and motivation (perception of risk about the disease and confidence in the safety and efficacy of vaccines), according to the debate on strategies to combat disinformation about vaccines between the Brazilian Society of Immunology (SBIm) and IQC (Question of Science Institute).^15^

Regarding race, most patients/guardians declared themselves mixed (84.4%), which reflects the origin of the disease and the process of colonization of Tocantins.^3,4^ This information was recorded in all medical records. In order for the federal government to grant sickness benefits to chronic disease, the person must be part of a family with no registered income, or with an income of no more than one-fourth of the minimum wage per capita.^6^ Most of the data recorded in medical records on this item proves the social inequality suffered by such patients as a result of the slavery process in Brazil, despite the abolition of slavery, without due historical reparation, especially in the North and Northeast regions of the country.^1^

In terms of blood type, O+ (53.2% of the study population) and A+ (27.3%) were the most common. These data are in line with Brazilian studies of the predominant blood types in the SCD population.^16^

With regard to weather and climate issues, 74 (48.1%) patients/guardians reported that the climate interferes with their illness, especially the worsening of pain symptoms with changes in the weather, especially the cold, or cold water/river bathing. These results are in line with the literature, as situations that lead to the body cooling down increase the possibility of crises, due to the peripheral vasoconstriction triggered by the cold.^2,17^

In addition to this issue of the relationship between pain and the cold, due to the pathophysiology of the disease, those responsible for pediatric sickle cell patients also reported coughing, getting sick with “thermal shock,” flu, respiratory allergies, fever, pneumonia, sore throat, rhinitis, and asthma, in association with changes in temperature, especially the cold, together with pain or not. These data indicate possible allergic and/or infectious diseases and are in line with the literature, especially as these sickle cell patients are part of risk groups for respiratory diseases aggravated by pollution, which include pediatric patients, pregnant women, the elderly, and people with comorbidities. Therefore, special care is needed for these patients, especially with preventive measures such as adequate body heating, good hydration, correct use of prescribed medications, regular cleaning of the house, a well-ventilated environment, and follow-up with a specialist.^18^

Because most sickle cell patients in Brazil are descendants of people from Africa, from where they were brought to work as slaves, and despite the abolition of slavery, they have received fewer opportunities for socioeconomic growth over the years. Therefore, patients with SCD generally live in poor areas, where there is environmental pollution, a lack of basic sanitation (or, if present, precarious sanitation), and inadequate quality of air, water, hygiene, and transportation. These environmental aspects contribute to the increased morbidity and mortality of sickle cell patients.^1^

The situation is exacerbated by the fact that the south and east of the Legal Amazon (which includes Tocantins) are part of the “arc of fire,” where fires and deforestation are rampant, which contributes to respiratory diseases, especially in the pediatric population, where SCD already predisposes them due to functional asplenia. Other health problems, which are increasing where there are anthropogenic processes promoting environmental changes and which make this population even more vulnerable, include infectious and parasitic diseases. Furthermore, endemic diseases such as visceral leishmaniasis and leprosy stand out in Tocantins, whose comorbidities in the SCD can cause sequelae and even lethality, with overlapping risks between them.^19^

Bearing in mind that changes in climate (including contact with different climates) and weather (temperature) also aggravate allergies, this theme was chosen for World Allergy Week in 2023, highlighting its relevance.^18^ Patients with SCD are not exempt from this comorbidity; on the contrary, due to their exposure to the aforementioned pollutants, they can manifest allergic diseases concomitantly with the symptoms of the underlying disease.

In addition to this factor, other studies have also shown that late diagnosis of SCD is related to death in young patients,^20^ while the opposite is also true: early diagnosis is essential for public health, reducing infant mortality, and the need for hospitalizations.^3^ It is therefore important to monitor this condition from an early age, starting with neonatal screening (heel prick test), with follow-up care preferably in specialized reference centers that include comprehensive, multi-professional, and interdisciplinary care.^21^

Within this holistic patient care, there are opportunities beyond “conventional medicine,” according to recommendations from the World Health Organization (WHO), which classifies what it calls “traditional medicine” and “complementary and alternative medicine” as “complementary integrative practices,” recommending that its member states develop national policies on the subject. In line with this recommendation, Brazil has developed the “National Policy for Integrative and Complementary Practices” (PNPIC) in the SUS^22^ and, based on this, other policies, programs, and projects have emerged, in particular the “National Program for Medicinal Plants and Phytotherapeutics.”^23^

In this context, “medicinal plant” is defined as a plant species used for therapeutic purposes, and “herbal medicine” as a product obtained from the medicinal plant, or its derivatives, for prophylactic, curative, or palliative purposes.^23^

It is therefore worth noting the value of studies that contribute to the therapeutic identification of flora and also pay attention to possible undesirable effects on patient safety,^24^ especially in the pediatric population, due to the peculiarity and sensitivity characteristic of this age group, which leads to the limitation of research in this area.^2^

As for the medications used by patients, the following stand out: Folic acid, prescribed due to the high cellular turnover of hemolysis in SCD, that is, there is a need for a greater quantity of substrate available in the body, so as not to compromise the renewal of red blood cells; dipyrone and paracetamol, the main analgesics and antipyretics used in the pediatric age group; phenoxymethylpenicillin, used for antibiotic prophylaxis in patients up to five years of age; and hydroxyurea, used to increase fetal hemoglobin in patients, which prevents crises of SCD, improving their quality of life. These findings corroborate the Ministry of Health’s manuals and protocols for the treatment of SCD.^25,26,27^

It should also be noted that 95.5% of the medical records reported the use of at least one medication (folic acid) at the last consultation, while 70.1% reported the use of medicinal plants. This shows that the majority of patients in this study use both conventional and traditional and/or complementary and alternative medicine. Considering the multi-professional care that these patients receive in this outpatient clinic, and the openness to understanding the cultural reality of this public, this could be a first step toward practicing the recommendations of the WHO and the Ministry of Health.^22,23^

Of the 64 plants cited in the medical records of pediatric sickle cell patients, only 23 were found in a literature review of medicinal plants used in SCD, although not for the same purpose.^8^ Of the six plants cited for the specific treatment of SCD, only one (onion) was found in the literature for this purpose,^28,29^ so the others are objects of investigation for further studies.

The main limitations of this research lie in the fact that patients/family members do not always report the use of other treatments to the health team, and/or the team does not always record such use in the medical records, the possibility of information bias, typical of this type of study, in addition to the loss of follow-up of some patients, who did not attend the service for more than 2 years, especially after the COVID-19 pandemic, causing their medical records to be archived and, therefore, it was not possible to include them in this study.

This research highlights the need for more research into medicinal plants for pediatric patients with SCD, as there is little mention of this age group in the scientific literature. It also highlights the importance of investing in the popular pharmacopoeia of the northern region of Brazil for this disease.

As for the use of medicinal plants mentioned in the medical records reviewed in relation to pediatric patients with SCD in Tocantins, lemon vine/*Ora-pro-nóbis* (*Pereskia aculeata*) stands out in the reports for the disease itself, requiring further studies to test whether its properties are in fact anti-sickle cell or not, and how this belief came to be that it can be helpful in the treatment. As for symptoms associated with the disease, the plants used for flu symptoms/COVID-19 stand out and need to be checked for these and other purposes.

Our findings also highlight the need for better coordination between conventional medicine and integrative and complementary practices, especially in pediatrics, taking into account the cultural use of medicinal plants by these families, without disregarding possible drug interactions with the drugs that this public uses for their underlying disease and their consequences and, above all, valuing the safety of these children or adolescents. Finally, the results reveal a low use of hydroxyurea in this population, that is, patients are not effectively receiving the most important, proven effective treatment for their disease, so the causes and difficulties in expanding access to this medication need to be clarified. A medication that significantly reduces the morbidity and mortality of the disease must have its use encouraged and expanded, and this is a suggestion for future studies.

## Data Availability

The database that originated the article is available with the corresponding author.
